# Research on High-Temperature Damage Behavior of Super Martensitic Stainless Steel 04Cr13Ni5Mo Based on Finite Element Simulation

**DOI:** 10.3390/ma18235262

**Published:** 2025-11-21

**Authors:** Tonghui Sun, Jihong Tian, Huiqin Chen, Bo Zhang, Fei Li, Hongqiang Shi

**Affiliations:** 1 School of Materials Science and Engineering, Taiyuan University of Science and Technology, Taiyuan 030024, China; sunth2009@126.com (T.S.); tykdtjh@tyust.edu.cn (J.T.); 2021002@tyust.edu.cn (F.L.); 2 Luoyang CITIC HIC Casting & Forging Co., Ltd., Luoyang 471000, China; zhangbo32103@126.com (B.Z.); 18703855514@163.com (H.S.); 3 CITIC Heavy Industries Co., Ltd., Luoyang 471000, China

**Keywords:** 04Cr13Ni5Mo, martensitic stainless steel, damaging behavior, high-temperature damage model, finite element simulation

## Abstract

04Cr13Ni5Mo martensitic stainless steel is extremely sensitive to forging temperature and is prone to generating extremely large cracks, which leads to the failure of forging. Therefore, high-temperature tensile tests were performed on 04Cr13Ni5Mo martensitic stainless steel using a Gleeble-1500D thermo-mechanical simulator to investigate its damage mechanisms. The tests covered a temperature range of 950–1200 °C and strain rates of 0.001–1 s^−1^. The high-temperature damage behavior and tissue evolution law at high temperatures were studied by means of EBSD, TEM, etc. Secondly, two high-temperature damage models of 04Cr13Ni5Mo, namely Normalized Cockcroft and Latham (NCL) and Oyane, were established by combining optimization algorithm and finite element simulation. Then, the two high-temperature damage models were integrated into the Forge^®^NxT 3.2 finite element software. Simulated thermal tensile tests were conducted on 04Cr13Ni5Mo at temperatures from 950 to 1200 °C, strain rates from 0.001 to 1 s^−1^. A comparison was made between the predicted and experimentally measured fracture displacements of the tensile specimens. The calculated correlation coefficients (*R*) were 0.995 and 0.991, respectively. It was determined that the NCL model has better simulation accuracy for predicting the forging cracks of 04Cr13Ni5Mo. The reliability of the finite element method for predicting forging crack defects in 04Cr13Ni5Mo forgings was established.

## 1. Introduction

Hydropower has emerged as a leading source in the development and utilization of renewable energy [1]. Its operating principle is to harness the energy of flowing water, converting it into mechanical energy via the turbine runner, which then drives the generator for electricity production. Common turbine types include impulse and Francis turbines [2,3]. Among these, impulse turbines are characterized by a wide effective load range and serve as the primary model for harnessing high-head hydropower resources [4]. As the core energy conversion component, the runner is subjected to multiple complex flow impact forces and plays a decisive role in both energy conversion efficiency and service life [5,6]. Often referred to as the “heart” of the turbine, it operates under extremely harsh conditions—withstanding high impact forces, sand erosion, low-temperature environments, and other challenges [7]—which impose stringent requirements on material properties such as corrosion resistance, low-temperature impact toughness, and microstructural homogeneity. Ultra-low carbon martensitic stainless steel, known for its excellent comprehensive mechanical properties, has become the preferred material for large impulse runners [8]. However, owing to its high deformation resistance and limited forging temperature window, cracks are prone to initiate and propagate during forging, leading to material waste. Hence, it is essential to investigate the high-temperature damage mechanism, establish a reliable damage model, and predict cracking behavior using finite element simulation.

During the plastic deformation of martensitic stainless steel, complex microstructural evolution occurs, which ultimately influences the performance of the forged components. Several scholars have conducted relevant studies in this area. Chen et al. [9] found that within a strain rate range of 10–10^3^ s^−1^, only dislocation motion and α′-martensite transformation were activated, resulting in the formation of nanocrystals and ultrafine grains. Salar Salahi et al. [10] examined the influence of deformation on both the microstructural evolution and corrosion performance of AISI 420. Specimens were cold-rolled to 25% and 50% deformation, revealing that the base material possessed significantly improved corrosion performance, marked by a more stable passivation layer.

Meysam Naghizadeh et al. [11] investigated the microstructural evolution during the annealing of deformed AISI 304. Their results indicated that the primary recrystallization of retained austenite delayed the development of an equiaxed microstructure, primarily through the coarsening of fine reversed grains. Liu et al. [12] examined the microstructure of Cr13 under different heat treatments. The quenched structure consisted mainly of lath martensite with a little retained austenite.

During the plastic processing of martensitic stainless steel, factors such as load, temperature, strain, and stress induce microstructural changes. Ductile damage and fracture behavior have attracted extensive research attention. Song et al. [13] developed a reliable predictive model for low-cycle fatigue crack propagation based on the framework of cumulative plastic damage. Chen et al. [14] optimized Ti6Al4V damage parameters until the objective function was minimized, thereby obtaining optimal damage model parameters. These high-temperature damage models were embedded into Forge^®^ to analyze damage evolution. Liu et al. [15] proposed a novel damage model by the Bonora model. Using a genetic algorithm-based inverse method, they identified four damage parameters by correlating experimental and simulation data from high-temperature tensile tests at various strain rates, and expressed these parameters as functions of temperature and strain rate. Rong et al. [16] experimentally studied the thermomechanical behavior and forming limits of AA7075 alloy sheets. They developed a continuum damage constitutive equations (CDCEs) to describe the tensile behavior of AA7075. Jia et al. [17] investigated the fracture behavior of AZ31B through compression tests and numerical simulations across a range of temperatures and strain rates. They analyzed the strain rate and temperature sensitivity of fracture morphology and fracture criteria, and proposed that at low temperatures, the material is prone to microcrack initiation.

Computer-aided solution of damage models has become an efficient computational approach. Finite element simulation technology has been widely adopted to predict damage behavior. Alkayem et al. [18] summarized various residual measures between finite element model predictions and physical test data for constructing objective functions in damage tracking. They addressed parameter selection in simulations model updating, proposed an evolutionary algorithm for damage detection. Felder et al. [19] employed a gradient extension, and validated a fully coupled multiphysics formulation, including strain localization and material and thermal softening. The model’s predictions were compared both quantitatively and qualitatively with experimental data, demonstrating its robustness and flexibility. Gao et al. [20] established a crystal plasticity finite element model incorporating microstructural features and damage nucleation mechanisms, revealing complex nucleation behaviors under microscale heterogeneous deformation.

Currently, with the planned construction of large-scale turbine generator units such as those at Zala and Yajiang in China, the size and performance requirements of impulse runner forgings are approaching material limits. Therefore, research on the high-temperature damage behavior of 04Cr13Ni5Mo for hydropower applications is of great importance. This paper investigates the high-temperature damage mechanism of 04Cr13Ni5Mo within the temperature of 950–1200 °C and strain rates of 0.001–1 s^−1^ through high-temperature tensile tests. An accurate and reliable high-temperature damage model is established and validated via simulation, providing theoretical support for the manufacturing of super-large runners.

## 2. Materials and Methods

**Figure 1:**
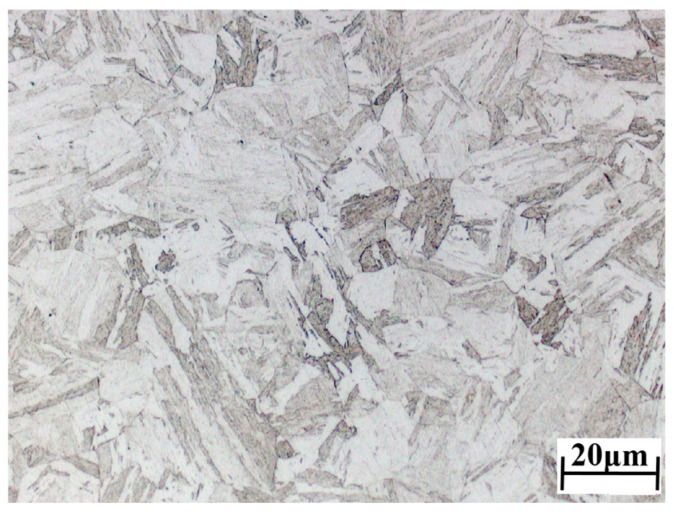
The original microstructure of 04Cr13Ni5Mo test materials.

**Figure 2:**
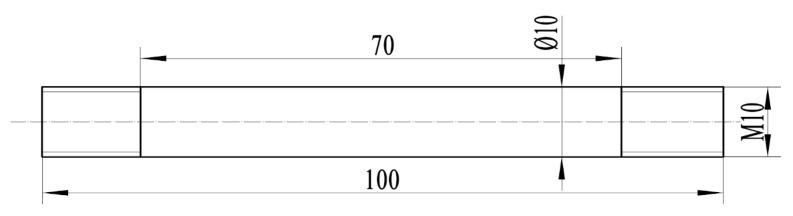
Diagram of tensile specimen size of 04Cr13Ni5Mo martensitic stainless steel.

**Figure 3:**
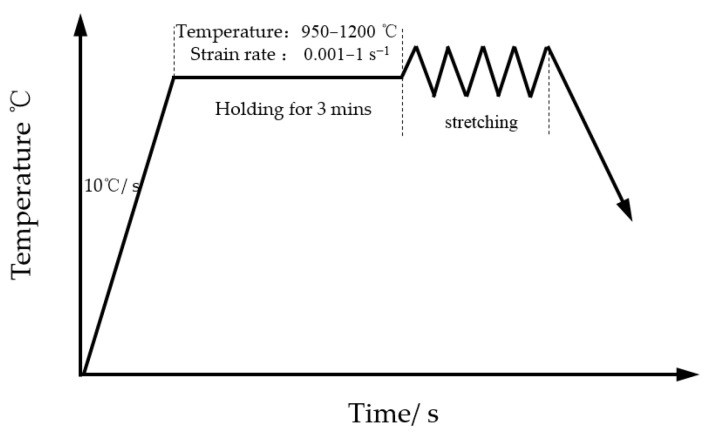
Schematic diagram of high-temperature tensile test of 04Cr13Ni5Mo martensitic stainless steel.

The 04Cr13Ni5Mo was selected as the subject material. Its initial microstructure is shown in Figure 1; the grain size is 26.81 μm. The chemical composition of 04Cr13Ni5Mo stainless steel (mass fraction, %) is given in Table 1, and the material performance parameters are shown in Table 2. High-temperature tensile testing of 04Cr13Ni5Mo was performed on a Gleeble 1500D thermo-mechanical simulation testing machine (Dynamic Systems Inc., New York, NY, USA), and as presented in Figure 2, the tensile specimens were fabricated with a geometry of φ10 × 100 mm. Due to the fact that 04Cr13Ni5Mo forgings are heated to 1200 °C when taken out of the furnace and then cooled to 950 °C for forging, a large number of cracks often occur. Moreover, the deformation speed of the forging press is relatively slow, and the general strain rate is below 1 s^−1^. A combination of tensile temperatures (950, 1000, 1050, 1100, 1150, and 1200 °C) and strain rates (0.001, 0.01, 0.1, and 1 s^−1^) was employed, as presented in Figure 3. The sample was heated to the target temperature (heating rate: 10 °C/s), held for 3 min, and then stretched at the set strain rate until it broke. Finally, test blocks were prepared at the fracture points of the specimens, and their microstructures were observed.

The 04Cr13Ni5Mo material high-temperature damage models were established and written into the Forge, and high-temperature tensile tests were carried out under the experimental conditions.

## 3. Results

### 3.1. High-Temperature Damage Behavior and Microstructure Evolution of 04Cr13Ni5Mo Martensitic Stainless Steel

**Figure 4:**
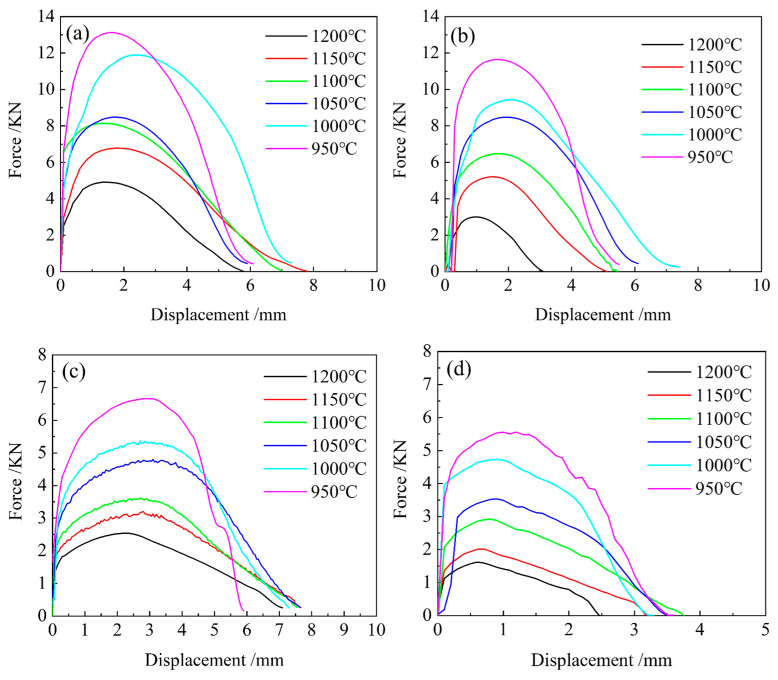
High-temperature tensile disposition–force curve of 04Cr13Ni5Mo martensitic stainless steel. (**a**) 1 s^−1^; (**b**) 0.1 s^−1^; (**c**) 0.01 s^−1^; (**d**) 0.001 s^−1^.

High-temperature tensile testing of 04Cr13Ni5Mo martensitic stainless steel produced the force–displacement curves shown in Figure 4. During thermal stretching until fracture, elastic deformation occurs only at very small strains (less than 0.01), which can be attributed to the decrease in yield strength of the material at elevated temperatures. With further stretching, plastic deformation becomes the dominant mechanism.

As presented in Figure 4a, at 1050 °C and strain rates ranging from 1 to 0.001 s^−1^, the peak tensile forces are 8.45, 8.42, 4.78, and 3.51 kN, respectively. These values indicate a positive correlation between peak tensile force and strain rate. As the strain rate decreases to 0.1 s^−1^, the peak force drops lightly by 0.03 kN. However, a further reduction to 0.01 s^−1^ leads to a pronounced decrease in peak force of approximately 43%, suggesting improved plasticity of 04Cr13Ni5Mo martensitic stainless steel at lower strain rates.

Figure 4b shows the results at a strain rate of 1 s^−1^ over the temperature range of 950–1200 °C. The peak tensile forces are 11.59, 9.44, 8.69, 6.52, 5.18, and 2.97 kN, respectively, with corresponding strain values at peak force being 1.81, 2.12, 1.97, 1.73, 1.47, and 0.96. Under constant strain rate, the temperature is negatively correlated with the peak tensile force and positively correlated with the peak strain. This implies that the optimal deformation temperature for 04Cr13Ni5Mo martensitic stainless steel lies between 1000 and 1050 °C, and deviations from this range adversely affect its plasticity.

**Figure 5:**
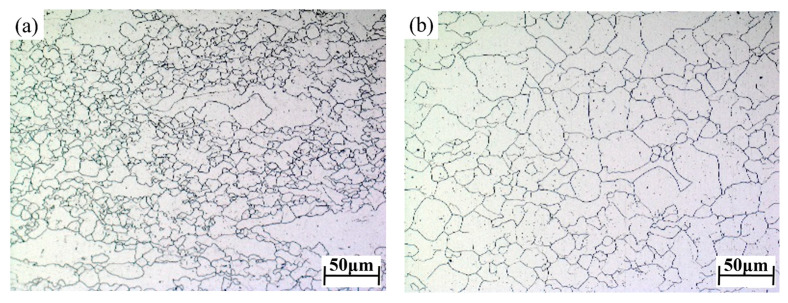

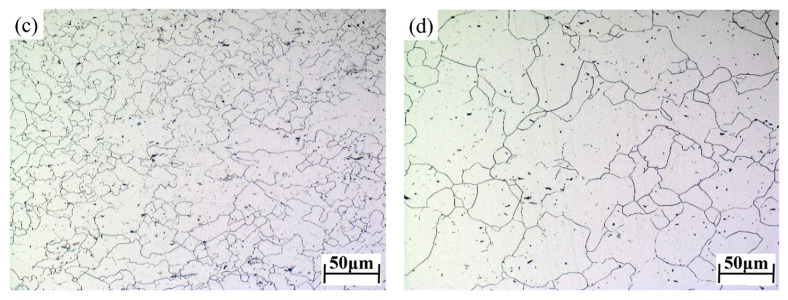
The microstructure of 04Cr13Ni5Mo under different conditions. (**a**) 1000 °C/0.1 s^−1^; (**b**) 1100 °C/0.1 s^−1^; (**c**) 1000 °C/0.01 s^−1^; (**d**) 1100 °C/0.01 s^−1^.

The damage behavior of 04Cr13Ni5Mo stainless steel under combined temperature and strain rate effects is controlled by its microstructural evolution. Figure 5 shows the microstructure near the tensile fracture of the specimens. At 1000 °C/0.01 s^−1^, dynamic recrystallization is incomplete, leaving some coarse grains unrefined, and a discernible amount of carbide precipitation was present. When the temperature increases to 1100 °C, grain uniformity improves, and the number density of the precipitated carbides decreased, with no significant change in their size distribution. However, at a higher strain rate (i.e., 1100 °C/0.1 s^−1^), the refined grains undergo coarsening, resulting in a uniform but relatively coarse microstructure, and no significant change was observed in the number of precipitated carbides; however, a distinct refinement in carbide size occurred.

**Figure 6:**
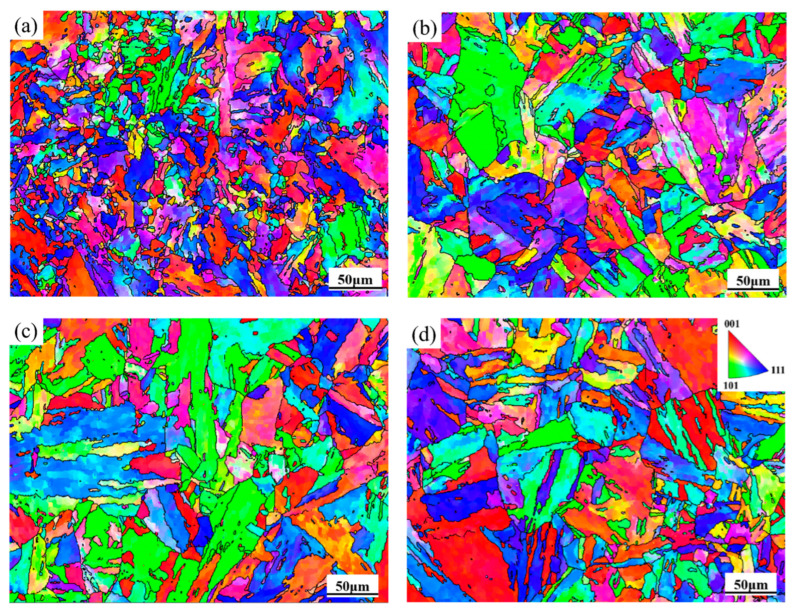
The IPF images of 04Cr13Ni5Mo. (**a**) 1000 °C/0.1 s^−1^; (**b**) 1100 °C/0.1 s^−1^; (**c**) 1000 °C/0.01 s^−1^; (**d**) 1100 °C/0.01 s^−1^.

The Inverse Pole Figure (IPF) in Figure 6 shows that the average grain size of 04Cr13Ni5Mo is 19.3 μm at 1000 °C/0.1 s^−1^ and 33.7 μm at 1100 °C/0.1 s^−1^. When the strain rate is 0.01 s^−1^, the average grain size is 28.4 μm at 1000 °C and 47.6 μm at 1100 °C.

**Figure 7:**
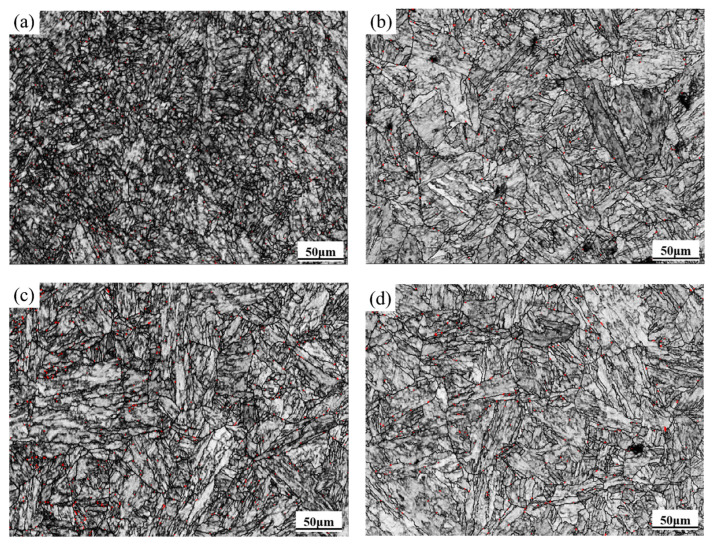
The phase distribution images (red indicates residual austenite) of 04Cr13Ni5Mo under different conditions. (**a**) 1000 °C/0.1 s^−1^; (**b**) 1100 °C/0.1 s^−1^; (**c**) 1000 °C/0.01 s^−1^; (**d**) 1100 °C/0.01 s^−1^.

According to the EBSD phase distribution results (Figure 7), a small amount of residual austenite (less than 0.5%) is present in the specimens, as indicated by the red regions in the figure. Specifically, the residual austenite content is 1% at 1000 °C/0.1 s^−1^ and 2% at 1000 °C/0.01 s^−1^. At 1100 °C, the content reaches 4% for both strain rates of 0.1 s^−1^ and 0.01 s^−1^. These results suggest that the residual austenite content increases with temperature but exhibits only a weak dependence on strain rate.

**Figure 8:**
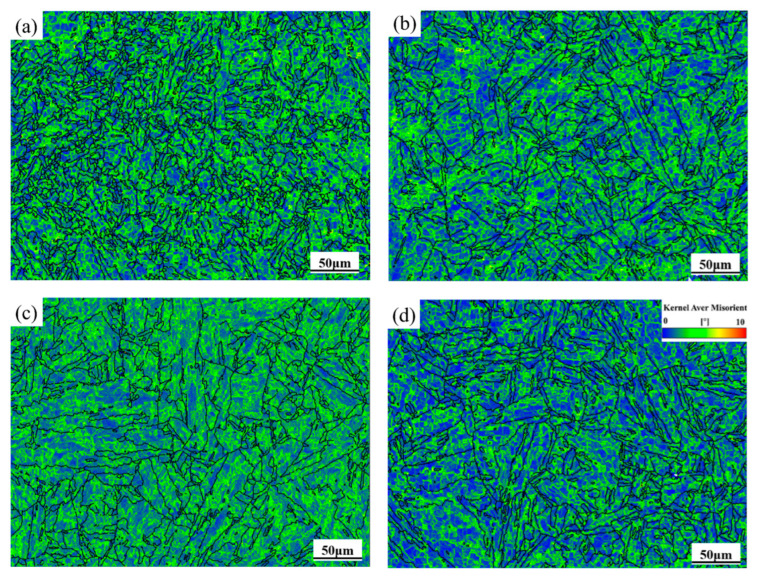
The KAM diagram of 04Cr13Ni5Mo. (**a**) 1000 °C/0.1 s^−1^; (**b**) 1100 °C/0.1 s^−1^; (**c**) 1000 °C/0.01 s^−1^; (**d**) 1100 °C/0.01 s^−1^.

The Kernel Average Misorientation (KAM) map serves as a qualitative indicator of the uniformity of plastic deformation in 04Cr13Ni5Mo steel. As can be seen from Figure 8, the dislocation density is the highest at 1000 °C/0.1 s^−1^, and the lowest at 1100 °C/0.01 s^−1^. This is because the material has fully completed recrystallization in high temperatures and low strain rates, resulting in a reduction in dislocation density.

**Figure 9:**
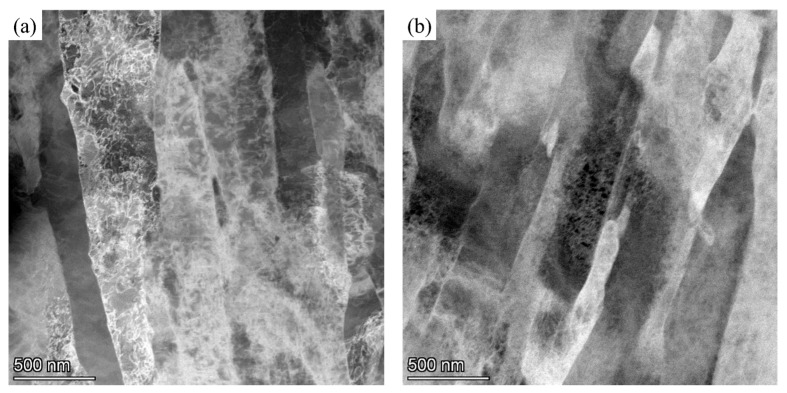
Typical STEM morphology images of 04Cr13Ni5Mo dislocation type martensite. (**a**) 1000 °C/0.1 s^−1^ dark field image at 20k magnification; (**b**) 1100 °C/0.1 s^−1^ dark field image.

Figure 9 presents the dark-field images of dislocation martensite under high magnification in a transmission electron microscope at a strain rate of 0.1 s^−1^ under temperature conditions of 1000 and 1100 °C. According to Figure 9a, the microstructure under 1000 °C/0.1 s^−1^ tensile deformation is filled with dislocation entanglements inside the martensite in the high-magnification dark-field image. As can be seen in Figure 9b, the tissue with tensile deformation at 1100 °C/0.1 s^−1^ shows a significant decrease in intrauterine entanglement.

### 3.2. High-Temperature Damage Behavior and Microstructure Evolution of 04Cr13Ni5Mo Martensitic Stainless Steel

#### 3.2.1. Establishment of a High-Temperature Damage Model for 04Cr13Ni5Mo Martensitic Stainless Steel

Oyane [21] proposed that cracks are caused by the connection of micro-pores in materials and put forward the corresponding model (Oyane damage model):

(1) $$C=\int_{0}^{{\bar{\varepsilon }}_{f}}(1+\alpha \sigma_{m}/\bar{\sigma })d\bar{\varepsilon }$$

In the formula, *C* represents the absolute damage value, $\sigma_{m}$ represents the hydrostatic stress, ${\bar{\varepsilon }}_{f}$ represents the fracture strain, $\bar{\sigma }$ represents the equivalent stress, $\bar{\varepsilon }$ represents the equivalent strain, and α represents the material coefficient.

(2) $$\sigma_{m}=F/A$$

In the formula, *F* represents the constant force acting perpendicularly on the surface of an object; *A* represents the force bearing area.

(3) $$\bar{\sigma }=\sqrt{\frac{{\left(\sigma_{1}-\sigma_{2}\right)}^{2}+{\left(\sigma_{2}-\sigma_{3}\right)}^{2}+{\left(\sigma_{3}-\sigma_{1}\right)}^{2}}{2}}$$

In the formula, $\sigma_{1}$, $\sigma_{2}$ and $\sigma_{3}$ are, respectively, the first principal stress, the second principal stress and the third principal stress.

(4) $$\bar{\varepsilon }=\sqrt{\frac{3\cdot \left[{\left(\varepsilon_{1}-\varepsilon_{2}\right)}^{2}+{\left(\varepsilon_{2}-\varepsilon_{3}\right)}^{2}+{\left(\varepsilon_{3}-\varepsilon_{1}\right)}^{2}\right]}{2}}$$

In the formula, $\varepsilon_{1}$, $\varepsilon_{2}$ and $\varepsilon_{3}$ are, respectively, the first principal strain, the second principal strain and the third principal strain.

M G Cockcroft [22] believed that fracture was related to tensile stress and proposed the corresponding damage model (Cockcroft and Latham damage model):

(5) $$C=\int_{0}^{{\bar{\varepsilon }}_{f}}\sigma^{*}/\bar{\sigma }d\bar{\varepsilon }$$

In the formula, $\sigma^{*}$ represents the maximum tensile stress.

Based on this, Oh et al. [23] considered the void growth theory and established the Normalized Cockcroft and Latham damage model (i.e., NCL damage model):

(6) $$C=\int_{0}^{{\bar{\varepsilon }}_{f}}\sigma_{1}/\bar{\sigma }d\bar{\varepsilon }$$

In the formula, $\sigma_{1}$ is the principal stress.

In conclusion, the classic damage criteria can all be expressed as some functional form of the stress component integral along the strain, as shown in the following formula:

(7) $$C=\int_{0}^{{\bar{\varepsilon }}_{f}}f\left(\sigma_{ij}\right)d\bar{\varepsilon }$$

In the formula, $f\left(\sigma_{ij}\right)$ is the stress-related function expression and $\sigma_{ij}$ is the stress component.

When $f\left(\sigma_{ij}\right)$ is respectively $1+\alpha \sigma_{m}/\bar{\sigma }$ and $\sigma_{1}/\bar{\sigma }$, Formula (4) can respectively represent the NCL and Oyane damage models.

The damage evolution equation is formulated as [24]

(8) $$\dot{\psi }=-\frac{Y}{S}\dot{\bar{\varepsilon }}$$

In the formula, $\dot{\psi }$ represents the damage evolution rate, *Y* represents the release rate of damage strain energy, *S* is the damage intensity parameter, and $\dot{\bar{\varepsilon }}$ is the equivalent plastic strain rate.

(9) $$\dot{\bar{\varepsilon }}=\frac{\bar{\varepsilon }}{\Delta t}$$

In the formula, $\bar{\varepsilon }$ represents the equivalent strain and Δt is the unit time.

*S* is dependent on both temperature and strain rate, and can be expressed as

(10) $$S=S(T,\dot{\bar{\varepsilon }})$$

By moving the negative sign in Formula (8) to the denominator,

(11) $$\dot{\psi }=\frac{Y}{S(T,\dot{\bar{\varepsilon }})}\dot{\bar{\varepsilon }}$$

By integrating Equation (11), the formula for the toughness damage variable can be obtained:

(12) $$\dot{\psi }=\int_{0}^{t}\dot{\psi }dt=\int_{0}^{t}\frac{Y}{S(T,\dot{\bar{\varepsilon }})}\dot{\bar{\varepsilon }}dt=\int_{0}^{{\bar{\varepsilon }}_{f}}\frac{Y}{S(T,\bar{\varepsilon })}d\bar{\varepsilon }$$

Here, *t* represents the deformation time of the material, s.

Replace *Y* in Equation (12) with a function of stress:

(13) $${\dot{\psi }}_{f}=\int_{0}^{{\bar{\varepsilon }}_{f}}\frac{f\left(\sigma_{ij}\right)}{S(T,\bar{\varepsilon })}d\bar{\varepsilon }$$

In the formula, ${\dot{\psi }}_{f}$ is the damage value when the material is damaged. According to Equations (12) and (13), the expression for the normalized damage model is derived as

(14) $$D=\frac{\dot{\psi }}{{\dot{\psi }}_{f}}=\int_{0}^{{\bar{\varepsilon }}_{f}}\frac{f\left(\sigma_{ij}\right)}{{\dot{\psi }}_{f}S(T,\bar{\varepsilon })}d\bar{\varepsilon }$$

Here, *D* represents the relative damage value. When *D* = 1, the material fails through ductile fracture, and the *S* function can be represented in the form of $S(T,\dot{\bar{\varepsilon }})=S(Z)$ in the form of the Zener–Holloman parameter. Therefore, the damage model can be written in the following form.

(15) $$D=\frac{\dot{\psi }}{{\dot{\psi }}_{f}}=\int_{0}^{{\bar{\varepsilon }}_{f}}\frac{f\left(\sigma_{ij}\right)}{{\dot{\psi }}_{f}S(Z)}d\bar{\varepsilon }=\int_{0}^{{\bar{\varepsilon }}_{f}}\frac{f\left(\sigma_{ij}\right)}{H(Z)}d\bar{\varepsilon }$$

Combining Equation (15) with each fracture criterion respectively, the NCL and Oyane high-temperature damage models are obtained as shown in (16) to (17):

(16) $$D=\int_{0}^{{\bar{\varepsilon }}_{f}}\frac{\sigma_{1}/\bar{\sigma }}{H_{1}(Z)}d\bar{\varepsilon }\;(\mathrm{NCL})$$

(17) $$D=\int_{0}^{{\bar{\varepsilon }}_{f}}\frac{1+\alpha \sigma_{m}/\bar{\sigma }}{H_{2}(Z)}d\bar{\varepsilon }\;(\mathrm{Oyane})$$

#### 3.2.2. Parameter Characterization for High-Temperature Damage Model of 04Cr13Ni5Mo Martensitic Stainless Steel

**Figure 10:**
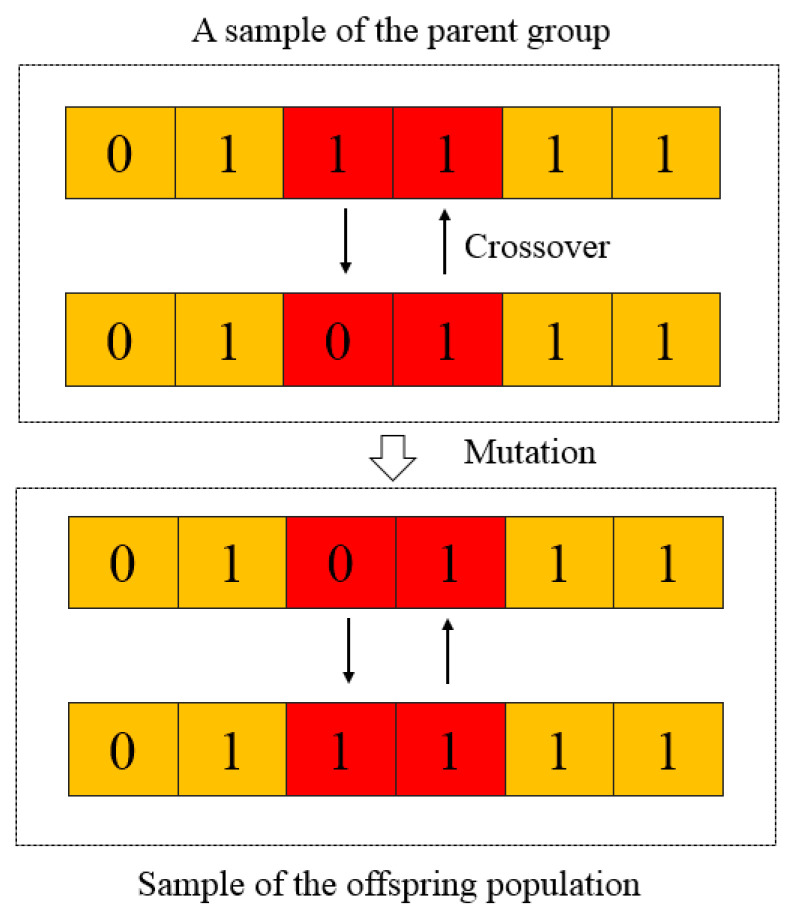
Genetic algorithm mutation and crossover structure diagram. The yellow part is directly inherited, the red part has undergone crossover and mutation.

Genetic algorithm (GA) is an optimization algorithm proposed by Professor Holland that simulates biological genetics and follows natural laws [25]. It solves the optimal solution by simulating the biological evolution process in nature. The basic principle is to convert the problem into chromosome encoding and manipulate the chromosomes through operations such as crossover and mutation, as shown in Figure 10, thereby obtaining a better solution and generating the optimal solution [26]. In this paper, the genetic algorithm is selected as the means of damage parameters to establish the numerical simulation of the tensile test. Through the FORTRAN computer programming language, the user secondary development interface of the finite element software is integrated with the optimization algorithm program to automatically optimize and simulate the tensile tests of materials and identify high-temperature damage parameters. The solution is carried out in Forge^®^NxT 3.2 software, and the displacement loads of the simulation and the test are compared. Then, the objective function is used to evaluate the deviation between them. The solution with the smallest deviation is the optimal one. The objective function is shown in Equation (18).

(18) $$\varphi =\frac{\sum_{i}\left[{\left(y_{i}^{\exp}-y_{i}^{num}\right)}^{2}\left(x_{i}-x_{i-1}\right)\right]}{\sum_{i}\left[{\left(y_{i}^{\exp}\right)}^{2}\left(x_{i}-x_{i-1}\right)\right]}$$

In the formula, ${y_{i}}^{\exp}$ is the load corresponding to the *i*-th data point in the test, ${y_{i}}^{\exp}$ represents the load corresponding to the *i*-th data point in the simulation, $x_{i}$ is the travel of the *i*-th data point, and $x_{i-1}$ is the travel of the (*i* − 1)-th data point. The closer the objective function φ is to 0, the closer the simulation result is to the experimental result.

**Figure 11:**
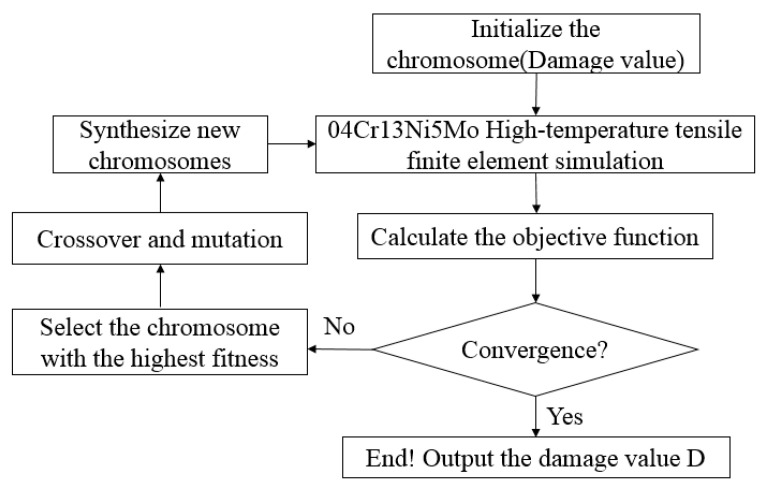
Procedure for identifying high-temperature damage model parameters.

Under different deformation conditions of high-temperature tensile testing of 04Cr13Ni5Mo stainless steel, the damage model parameters were identified by combining genetic algorithm and finite element method. The flowchart is shown in Figure 11. The genetic algorithm parameters are set as shown in Table 3. Then, finite element models were established in Forge^®^NxT 3.2 for tensile tests.

**Figure 12:**
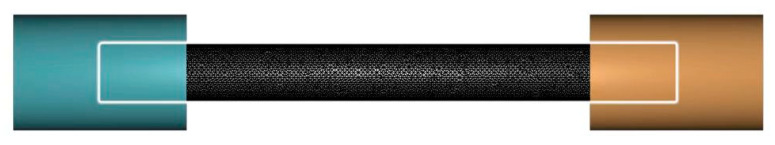
High-temperature tensile finite element simulation model of 04Cr13Ni5Mo.

A tensile finite element model was established in the Forge^®^ software, as shown in Figure 12, the finite element model adopts tetrahedral meshes, with a mesh size of 1mm and a total of 41,869 meshes, and tensile tests were conducted. To simulate the situation where the thread of the tensile specimen is clamped in the clamping fixture, the tensile specimen is extended into the clamping fixture by 15 mm. According to the actual experimental conditions, the specimens are tested under vacuum conditions without heat exchange with air, and the deformed specimens are heated to a specific test temperature under adiabatic conditions. Both ends of the sample are fixed to the left and right fixtures, it is set as a rigid body, and finally, simulation is conducted at the actual stretching speed.

The simulation adopts the Hansel–Spittel constitutive model. The model parameters are directly imported from the true stress and true strain data of the test and are automatically optimized and identified by the software, as shown in Equation (19):

(19) $$\sigma =374.247\cdot e^{-0.00875T}\cdot \varepsilon^{-0.028}\cdot {\dot{\varepsilon }}^{-0.2431}\cdot e^{\frac{-0.0024}{\varepsilon }}\cdot {\left(1+\varepsilon \right)}^{0.00427T}\cdot e^{-3.257\varepsilon }\cdot {\dot{\varepsilon }}^{-0.00017T}\cdot T^{1.104}$$

In the formula, σ represents the true stress, ε represents the true strain, $\dot{\varepsilon }$ represents the strain rate, and *T* represents the temperature.

The critical damage values *D*c of the Normalized Cockcroft and Latham and Oyane damage criteria of 04Cr13Ni5Mo stainless steel are shown in Table 4. The critical damage values of the two damage models vary due to their different damage theories. However, for 04Cr13Ni5Mo stainless steel, the variation trend of the critical damage value is similar. That is, when the temperature remains constant, a decrease in the strain rate will cause the critical damage value of the material to increase. At 950 to 1050 °C, the critical damage value increases with the rise in temperature. However, at 1100 °C, the critical damage value drops sharply, and as the temperature rises, the critical damage value decreases. This indicates that the plasticity of 04Cr13Ni5Mo is optimal at a low strain rate of around 1050° C. When the temperature continues to rise, its plasticity drops sharply instead.

The Zener–Hollomon parameter is a relationship expression that measures the influence of temperature and strain rate on thermal deformation behavior and has been widely applied in metallography. Equation (20) describes the relationship between temperature and strain rate [27]:

(20) $$Z=\dot{\varepsilon }\exp\left(\frac{Q}{RT}\right)$$

The interdependence of stress, temperature, and strain rate is governed by

(21) $$\dot{\varepsilon }=A[\sinh{\left(\alpha \sigma \right)}^{n_{2}}\exp(-Q/RT)]$$

In the formula, *Q* represents deformation activation energy/J·mol^−1^, *R* represents gas constant, *A* and *α* are material constant, and σ is stress/MPa.

Drawing on the test data of 04Cr13Ni5Mo stainless steel, a linear fit was performed on Equation (21), and $Q=621,473.316\;J\cdot {\mathrm{mol}}^{-1}$ was obtained. Therefore,

(22) $$Z=\dot{\varepsilon }\exp(\frac{621,473.316}{RT})$$

Quadratic term fitting was conducted on ln*Z* under different conditions and the critical damage values *D*c of damage criterion, resulting in

(23) $$H_{NCL}(Z)=-7.62455-0.24674\ln Z-0.00166{\left(\ln Z\right)}^{2}$$

(24) $$H_{\mathrm{Oyane}}(Z)=-8.34547+0.26684\ln Z-0.00179{\left(\ln Z\right)}^{2}$$

Then, the NCL high-temperature damage model of 04Cr13Ni5Mo stainless steel is obtained as

(25) $$\left\{\begin{array}{l} D=\int_{0}^{\varepsilon }\frac{\sigma_{1}/\bar{\sigma }}{-7.62455-0.24674\ln Z-0.00166{\left(\ln Z\right)}^{2}}d\bar{\varepsilon }\\ Z=\dot{\varepsilon }\exp(\frac{621,473.316}{RT}) \end{array}\right.$$

The Oyane high-temperature damage model is

(26) $$\left\{\begin{array}{l} D=\int_{0}^{\varepsilon }\frac{1+\alpha \sigma_{m}/\bar{\sigma }}{-8.34547+0.26684\ln Z-0.00179{\left(\ln Z\right)}^{2}}d\bar{\varepsilon }\\ Z=\dot{\varepsilon }\exp(\frac{621,473.316}{RT}) \end{array}\right.$$

To verify the accuracy of the established NCL and Oyane high-temperature damage models in predicting the damage and fracture of 04Cr13Ni5Mo stainless steel, and to determine the model that is most suitable for predicting the damage behavior of 04Cr13Ni5Mo stainless steel. The model was embedded in the finite element software Forge^®^ to conduct finite element numerical simulation of the uniaxial tensile test of 04Cr13Ni5Mo stainless steel at 950 °C–1200 °C, 0.001 s^−1^–1 s^−1^ conditions, and the experimental data were compared with simulation results to validate the model.

The test and simulated fracture displacements were determined using Equations (27) and (28), which calculate the difference between the initial and fracture lengths of the tensile specimen. The correlation coefficient *R* is calculated by Equation (29):

(27) $$\Delta L_{e}=L_{e}-L$$

(28) $$\Delta L_{s}=L_{s}-L$$

(29) $$R=\frac{\sum_{i-1}^{N}\left(\Delta L_{e}^{i}-\bar{\Delta L_{e}}\right)\left(\Delta L_{s}^{i}-\bar{\Delta L_{s}}\right)}{\sqrt{{\sum_{i=1}^{N}\left(\Delta L_{e}^{i}-\bar{\Delta L_{e}}\right)}^{2}}\sqrt{{\sum_{i=1}^{N}\left(\Delta L_{s}^{i}-\Delta L_{s}\right)}^{2}}}$$

In the formula, *L* represents the initial length of the specimen, *L_e_* represents the length of the specimen at the test fracture, *L_s_* represents the length of the specimen at the simulated fracture, *N* represents the total number of tensile test specimens, $\bar{L_{s}}$ represents the average value of *L_s_*, $\bar{L_{e}}$ represents the average value of *L_e_*, $\Delta L_{e}^{i}$ represents the *i*-th test fracture displacement, and $\Delta L_{s}^{i}$ represents the *i*-th simulated fracture displacement.

**Figure 13:**
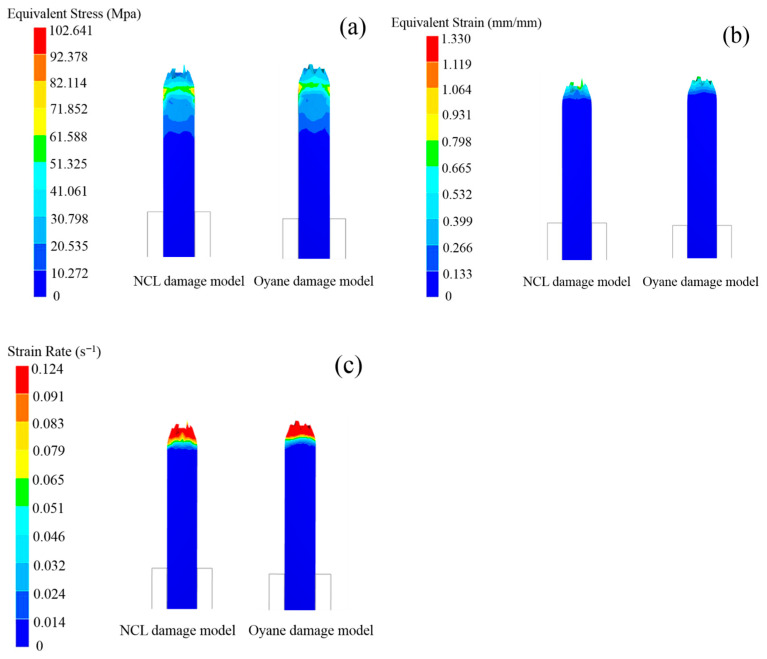
Comparison of the NCL and Oyane high-temperature damage models at 1000 °C/0.1 s^−1^. (**a**) equivalent stress; (**b**) equivalent strain; (**c**) strain rate.

As illustrated in Figure 13, the NCL and Oyane high-temperature damage models yield similar trends in equivalent stress, equivalent strain, and strain rate at tensile fracture. Both models converge in predicting that the location of maximum equivalent stress coincides with the onset of necking. Quantitatively, the NCL model predicts a higher maximum stress (92.378 MPa) than the Oyane model (82.114 MPa). Similarly, for equivalent strain, both models identify the fracture surface as the area of maximum concentration, with the NCL model again predicting a higher value (1.064) compared to Oyane’s 0.798. Furthermore, the simulation results indicate that both models predict a strain rate range of 0.091 to 0.124 s^−1^ from the necking zone to the fracture surface.

**Figure 14:**
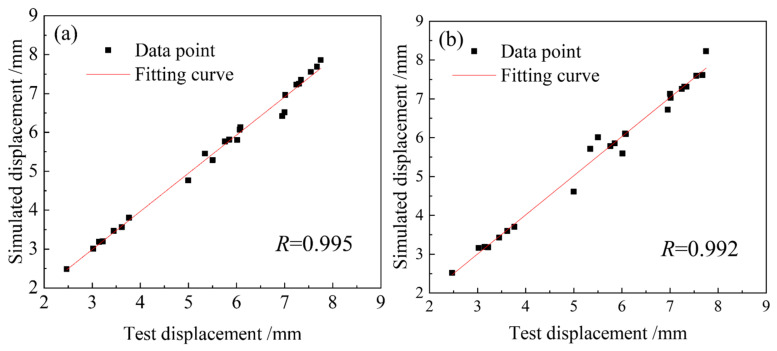
Correlation between model predictions and experimental data. (**a**) NCL damage model; (**b**) Oyane damage model.

**Figure 15:**
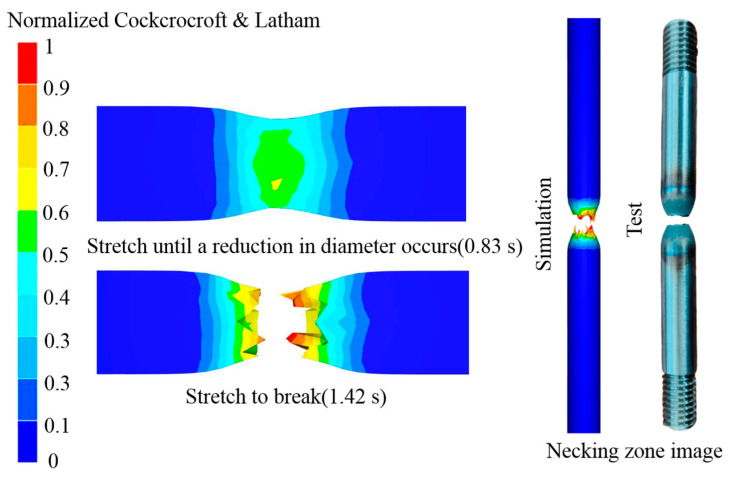
Tensile simulation results of 04Cr13Ni5Mobu stainless steel at 1000 °C/0.1 s^−1^.

**Figure 16:**
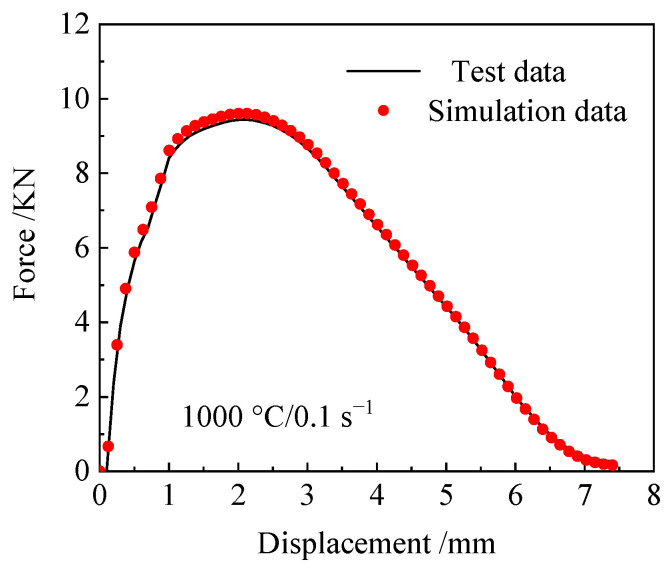
Simulation and test tensile force–displacement diagram at 1000 °C/0.1 s^−1^.

Figure 14 demonstrates a strong linear correlation between the simulated and experimental results. The correlation coefficients *R* of the two models, NCL and Oyane, are 0.995 and 0.991, respectively. It can be seen that both have high accuracy in predicting the fracture length of 04Cr13Ni5Mo stainless steel. However, the NCL high-temperature damage model is more reliable. Figure 15 shows the tensile simulation results of 04Cr13Ni5Mo stainless steel at 1000 °C/0.1 s^−1^. It can be seen that when the material is stretched for 0.83 s and its diameter decreases, about to develop cracks, the damage value on its outer surface is approximately 0.3, and the maximum damage value at the center is about 0.7. This indicates that during the process of cracking in 04Cr13Ni5Mo stainless steel, tiny holes first appear inside and gradually extend to fracture. When the material is stretched for 1.42 s and the damage value reaches 1, cracks appear in the sample and it is pulled apart. A comparative analysis of the necked area shows little difference in the fracture surface shape between the simulation and physical tests, thereby demonstrating the reliability of the simulated material fracture surface. A comparison between the simulated and experimental tensile force–displacement curves at 1000 °C/0.1 s^−1^ is presented in Figure 16. The close agreement between them, evidenced by a mere 4.38% maximum deviation, confirms the high accuracy of the simulation model.

## 4. Conclusions

To effectively predict cracking during the forging of 04Cr13Ni5Mo stainless steel super-large runners, this study conducted hot tensile tests on 04Cr13Ni5Mo stainless steel at temperatures from 950 to 1200 °C and strain rates from 0.001 to 1 s^−1^. The high-temperature damage and microstructure evolution behavior of the steel were analyzed, and high-temperature damage models based on Normalized Cockcroft and Latham and Oyane criteria were established.

The plasticity of 04Cr13Ni5Mo stainless steel improves with decreasing strain rate. Within the temperature from 1000 to 1050 °C, the material maintains relatively high plasticity. Excessively high temperatures favor the retention of austenite, leading to a significant drop in plasticity, while excessively low temperatures result in insufficient dynamic recrystallization, also reducing plasticity.

By integrating a genetic algorithm with finite element analysis to identify the parameters of the high-temperature damage model, the following expressions were established for 04Cr13Ni5Mo stainless steel:

$$\begin{array}{cc} \left\{\begin{array}{l} D=\int_{0}^{\varepsilon }\frac{\sigma_{1}/\bar{\sigma }}{-7.62455-0.24674\ln Z-0.00166{\left(\ln Z\right)}^{2}}d\bar{\varepsilon }\\ Z=\dot{\varepsilon }\exp(\frac{621,473.316}{RT}) \end{array}\right. & \left(\mathrm{NCL}\right) \end{array}$$

$$\begin{array}{cc} \left\{\begin{array}{l} D=\int_{0}^{\varepsilon }\frac{1+\alpha \sigma_{m}/\bar{\sigma }}{-8.34547+0.26684\ln Z-0.00179{\left(\ln Z\right)}^{2}}d\bar{\varepsilon }\\ Z=\dot{\varepsilon }\exp(\frac{621,473.316}{RT}) \end{array}\right. & \left(\mathrm{Oyane}\right) \end{array}$$

Both high-temperature damage models were embedded into Forge^®^ software for finite element simulation. Comparison with experimental high-temperature tensile fracture displacements was performed, and the accuracy of the models was quantified by means of the correlation coefficient (*R*). The correlation coefficients for the Normalized Cockcroft and Latham and Oyane high-temperature damage models were 0.995 and 0.991. This demonstrates that the damage models established using the optimization algorithm combined with finite element technology are highly reliable. For predicting the hot damage behavior of 04Cr13Ni5Mo stainless steel, the Normalized Cockcroft and Latham model exhibits greater applicability.

## Figures and Tables

**Table 1:** Chemical composition (wt.%) of the 04Cr13Ni5Mo steel.

Chemical Composition	Fe	C	Si	Mn	S	P	Cr	Ni	Mo	Cu
04Cr13Ni5Mo	80.423	0.033	0.36	0.71	0.003	0.018	12.9	4.9	0.6	0.053

**Table 2:** Performance parameter table of 04Cr13Ni5Mo material.

Parameter	Young Modulus/Gpa	Strength Stress/MPa	Tensile Strength/MPa	Hardness/HBW
04Cr13Ni5Mo	201	657	806	307

**Table 3:** Genetic algorithm configuration.

Population Size	Crossover Rate	Mutation Rate	Target Function Value	Maximum Iterations
25	0.8	0.05	0.03	150

**Table 4:** The critical damage values of each damage criterion of 04Cr13Ni5Mo.

Damage Criterion	Deformation Condition	950 °C	1000 °C	1050 °C	1100 °C	1150 °C	1200 °C
Normalized Cockcroft and Latham	0.001 s^−1^	1.573	1.692	1.861	1.342	1.307	1.107
	0.01 s^−1^	1.534	1.663	1.816	1.214	1.211	1.085
	0.1 s^−1^	1.484	1.562	1.748	1.184	1.164	0.987
	1 s^−1^	1.457	1.529	1.717	1.143	1.116	0.957
Oyane	0.001 s^−1^	1.604	1.687	1.833	1.360	1.297	1.061
	0.01 s^−1^	1.579	1.709	1.800	1.253	1.226	1.070
	0.1 s^−1^	1.448	1.582	1.762	1.172	1.195	0.996
	1 s^−1^	1.497	1.579	1.746	1.136	1.163	0.948

## Data Availability

The original contributions presented in this study are included in the article. Further inquiries can be directed to the corresponding author.
